# Bone morphogenetic protein 4 (BMP4) alleviates hepatic steatosis by increasing hepatic lipid turnover and inhibiting the mTORC1 signaling axis in hepatocytes

**DOI:** 10.18632/aging.102552

**Published:** 2019-12-12

**Authors:** Qi Peng, Bin Chen, Hao Wang, Ying Zhu, Jinghong Wu, Yetao Luo, Guowei Zuo, Jinyong Luo, Lan Zhou, Qiong Shi, Yaguang Weng, Ailong Huang, Tong-Chuan He, Jiaming Fan

**Affiliations:** 1 Ministry of Education Key Laboratory of Diagnostic Medicine, and School of Laboratory Medicine, Chongqing Medical University, Chongqing 400016, China; 2 Molecular Oncology Laboratory, Department of Orthopaedic Surgery and Rehabilitation Medicine, The University of Chicago Medical Center, Chicago, IL 60637, USA; 3 Clinical Epidemiology and Biostatistics Department, Department of Pediatric Research Institute, Children’s Hospital of Chongqing Medical University, Chongqing 400014, China; 4 Key Laboratory of Molecular Biology for Infectious Diseases of The Ministry of Education of China, Institute for Viral Hepatitis, Department of Infectious Diseases, The Second Affiliated Hospital of Chongqing Medical University, Chongqing, China

**Keywords:** non-alcoholic fatty liver disease (NAFLD), hepatic lipid metabolism, BMP signaling, mTORC1 signaling

## Abstract

Liver has numerous critical metabolic functions including lipid metabolism, which is usually dysregulated in obesity, the metabolic syndrome, and non-alcoholic fatty liver disease (NAFLD). Increasing evidence indicates bone morphogenetic proteins (BMPs) play an important role in adipogenesis and thermogenic balance in adipogenic progenitors and adipose tissue. However, the direct impact of BMPs on hepatic steatosis and possible association with NAFLD are poorly understood. Here, we found that BMP4 was up-regulated in oleic acid-induced steatosis and during the development of high fat diet (HFD)-induced NAFLD. Exogenous BMP4 reduced lipid accumulation and up-regulated the genes involved in lipid synthesis, storage and breakdown in hepatocytes. Exogenous BMP4 inhibited hepatic steatosis, reduced serum triglyceride levels and body weight, and alleviated progression of NAFLD *in vivo*. Mechanistically, BMP4 overexpression in hepatocytes down-regulated most components of the mTORC1 signaling axis. Collectively, these findings strongly suggest that BMP4 may play an essential role in regulating hepatic lipid metabolism and the molecular pathogenesis of NAFLD. Manipulating BMP4 and/or mTORC1 signaling axis may lead to the development of novel therapeutics for obesity, metabolic syndrome, and NAFLD.

## INTRODUCTION

Liver is an essential organ in carrying out critical metabolic functions, ranging from metabolism of nutrients, synthesis of glucose and lipids, to detoxification of drugs and xenobiotics. Disruptions of liver metabolic functions may lead to a broad range of liver diseases such as metabolic syndrome, obesity and cancer [1–4]. In fact, non-alcoholic fatty liver disease (NAFLD) resulted from obesity and the metabolic syndrome is the most common liver disease in the western world [1–4]. The pathological characteristics of NAFLD is the accumulation of triglycerides (TG) within hepatocytes (also known as, hepatic steatosis) [1–4]. Hepatic lipid metabolism is tightly regulated by hormones (such as insulin), nuclear receptors, numerous cellular signaling pathways and transcription factors [1], although molecular mechanisms through which hepatic lipid metabolism are regulated remain to be fully understood.

Bone morphogenetic proteins (BMPs) belong to the TGF-β superfamily, and play important roles in regulating embryonic development, stem cell differentiation, and adult tissue homeostasis [5–9]. There are at least 14 types of BMPs in rodents and humans [6, 7, 10]. Through a comprehensive analysis of the 14 types of human BMPs, we have demonstrated that several osteogenic BMPs, such as BMP2, 4, 6, 7 and 9, are potent factors to drive adipogenic differentiation of mesenchymal stem cells [10–13]. We and others further showed that BMP7 and BMP4 may act as a molecular switch in regulating white vs. brown adipogenesis and energy expenditure [14–20]. Furthermore, BMP7 was shown to reverse obesity phenotype by regulating appetite through the central mTOR pathway in the brain [14]. However, the effect of BMPs on lipogenesis, metabolism and energy expenditure has been mostly investigated in adipogenic progenitors and adipose tissue. Thus, the direct impact of BMP signaling on hepatic metabolism, especially on glucose and lipid production, as well as possible association of BMP signaling with NAFLD, remains to be fully understood.

Here, we demonstrated that exogenous BMP4 inhibited the hepatic steatosis, lowered serum triglyceride (TG) and body weight, and alleviated the development and progression of NAFLD in a mouse model. We further demonstrated that BMP4 exerted the above effects by promoting lipid turnover through up-regulating the genes involved in lipid metabolism, while suppressing mTORC1 signaling pathway both *in vitro* and *in vivo*. These results strongly suggest that BMP4 may play an essential role in regulating hepatic lipid metabolism and should aid us to understand the molecular pathogenesis of NAFLD. It is conceivable that manipulating BMP4 and/or mTORC1 signaling networks would lead to the development of novel therapeutics for obesity, metabolic syndrome, and NAFLD.

## RESULTS

### BMP4 is up-regulated in Oleic acid-induced steatosis and during the development of a mouse model of NAFLD

**Figure 1:**
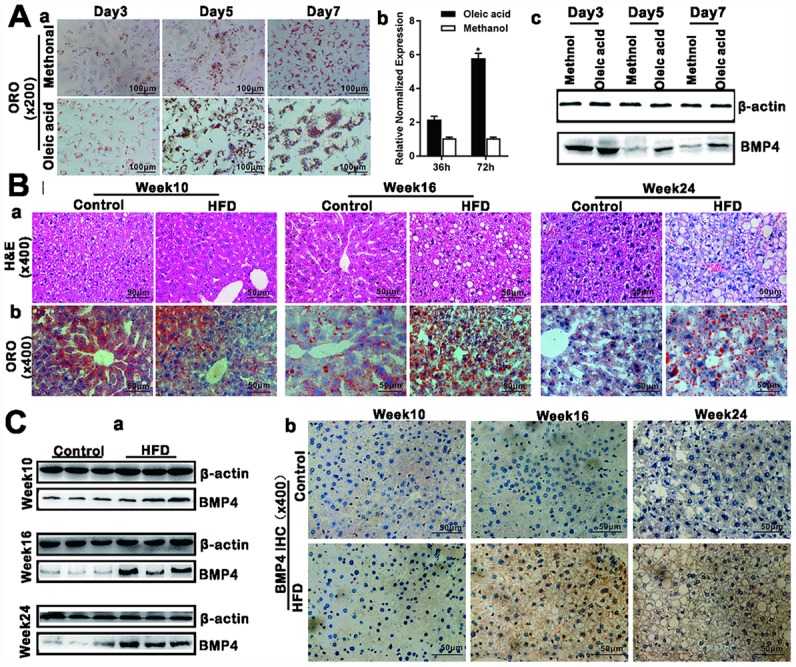
**BMP4 expression is elevated during Oleic acid-induced triglyceride/lipid accumulation in hepatocytes and in a mouse model of NAFLD.** (**A**) BMP4 expression in Oleic acid-induced lipid accumulation. Primary mouse hepatocytes were stimulated with 0.05mM Oleic acid (methanol as a vehicle control). ORO staining was done at days 3, 5 and 7 respectively. Representative images are shown (*a*). Alternatively, total RNA was isolated at 36h and 72h post Oleic acid treatment and subjected to TqPCR analysis of Bmp4 expression. Relative expression was calculated by dividing the relative expression values (i.e., *Bmp4*/*Gapdh*) in “*” p < 0.05, Oleic acid group vs. methanol group (*b*). Total protein was isolated and subjected to Western blotting analysis of BMP4 expression at days 3, 5 and 7 post Oleic acid treatment (*c*). (**B**) Establishment of the mouse model of NAFLD. C57/B6 mice (4-week-old male, n=10 /time point/group) were fed with 45% high fat diet (HFD) or normal diet (Control), and sacrificed at weeks 10, 16 and 24, respectively. The retrieved liver tissue was subjected to H & E staining (*a*) and ORO staining (*b*). (**C**) BMP4 expression in mouse liver tissue of NAFLD. Total protein was isolated from the mouse liver tissue of the HFD and Control groups at weeks 10, 16 and 24 respectively, and subjected to Western blotting analysis of BMP4 expression. (*a*). IHC (immunohistochemical) staining of BMP4 expression was detected in the liver from the HFD and Control groups respectively (*b*). Each assay condition was done in triplicate, and representative images are shown or indicated by arrows.

Oleic acid can induce the triglyceride lipid accumulation significantly in cultured hepatocytes [21, 22]. We showed that 0.05mm Oleic acid effectively induced hepatic triglyceride/lipid accumulation in 4-week-old mouse primary hepatocytes starting from days 3 to 7 contrast with that of control methanol group (Figure 1A, a). TqPCR assay and Western blotting analysis revealed that BMP4 expression was up-regulated upon oleic acid treatment of the primary hepatocytes (Figure 1A, b and c). We next examined the expression status of BMP4 in NAFLD. As shown in Figure 1B, we successfully established the mouse model of HFD-induced NAFLD as confirmed by H & E staining (Figure 1B, a) and ORO staining (Figure 1B, b) in the liver samples derived from the HFD group at weeks 16 and 24, compared with that of control group. Using Western blotting analysis, we found BMP4 expression in the liver samples was significantly up-regulated in the HFD group at week 16 and 24, compared with that of the control group (Figure 1C, a). Immunohistochemical staining further confirmed that BMP4 expression was elevated in the HFD group at weeks 16 and 24, compared with that of the control group (Figure 1C, b). Control IgG of liver tissue was shown in Supplementary Figure 1D, a. These results suggest that BMP4 expression may be associated with the development of hepatic steatosis.

### Exogenous BMP4 inhibits triglyceride/lipid accumulation but plays a paradoxical role in regulating the expression of the genes involved in lipid metabolism in hepatocytes

**Figure 2:**
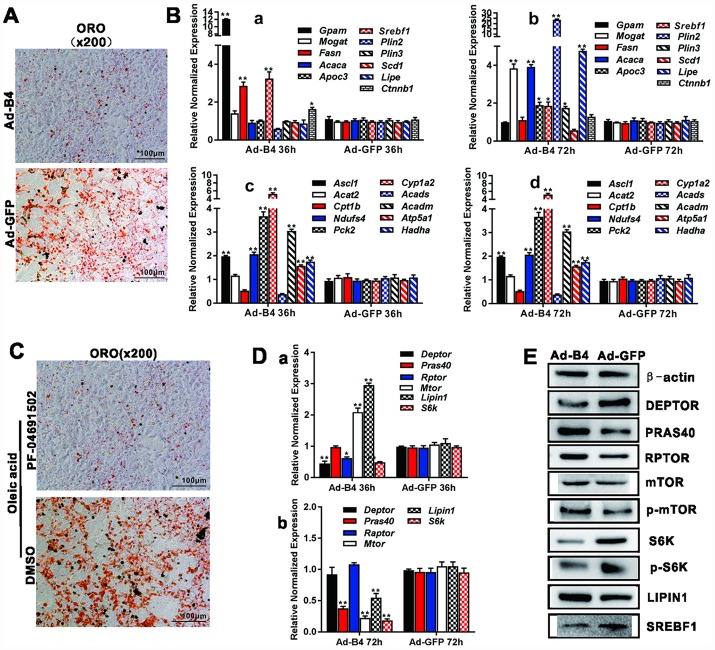
**BMP4 inhibits triglyceride accumulation through regulating the genes involved in lipid metabolism and members of mTORC1 signaling pathway in hepatocytes.** (**A**) Primary mouse hepatocytes were infected with Ad-B4 or Ad-GFP for 7 days, and subjected to ORO staining. (**B**) Primary mouse hepatocytes were infected with Ad-B4 or Ad-GFP for 36h and 72h. Total RNA was isolated and subjected to TqPCR analysis of the expression of the genes involved in triglyceride synthesis and storage (*a and b)* and triglyceride breakdown *(c and d)*. Relative expression was calculated by dividing the relative expression values (i.e., gene/*Gapdh*) in “**” p < 0.001, “*” p < 0.05, Ad-B4 group vs. Ad-GFP group. (**C**) Oleic acid (0.05mM)-induced hepatocytes were treated with 1nM PF-04691502 or DMSO for 7 days, and subjected to ORO staining. (**D**) Primary mouse hepatocytes were infected with Ad-B4 or Ad-GFP for 36h and 72h. Total RNA was isolated and subjected to TqPCR analysis of the expression of the members of mTORC1 signaling pathway (*a and b)*. Relative expression was calculated by dividing the relative expression values (i.e., gene/*Gapdh*) in “**” p < 0.01, “*” p < 0.05, Ad-B4 group vs. Ad-GFP group. (**E**) Primary mouse hepatocytes were infected with Ad-B4 or Ad-GFP for 72h, and total cell lysate was subjected to Western blotting analysis of the expression of the members of mTORC1 signaling pathway*.* Each assay condition was done in triplicate, and representative images are shown or indicated by arrows.

To effectively deliver and overexpress of BMP4 into cultured hepatocytes and liver tissue *in vivo*, we constructed the recombinant adenovirus Ad-B4and a mock control virus Ad-GFP. We demonstrated that Ad-B4 (and Ad-GFP) effectively infected primary hepatocytes and expresses a high level of BMP4 both at mRNA and protein levels (Supplementary Figure 1A and 1B). ORO staining showed that Ad-B4 significantly decreased hepatic triglyceride/lipid accumulation after 7 days, compared with that of Ad-GFP (Figure 2A). TqPCR results revealed that BMP4 up-regulated the expression of lipid synthesis and storage genes, such as *Gpam*, *Fasn*, *Srebf1* at 36h, and *Mogat*, *Acaca*, *Apoc3*, *Srebf1*, *Plin2*, *Lipe* at 72h (Figure 2B, a and b), as well as the expression of lipid breakdown genes, such as *Ascl2*, *Ndufs4*, *Cyp1a2*, *Acadm*, *Atp5a1*, *Hadha* at 36h, and *Ascl1*, *Ndufs4*, *Pck2*, *Cyp1a2*, *Acadm*, *Atp5a1*, *Hadha* at 72h (Figure 2B, c and d).

### Exogenous BMP4 inhibits hepatic lipid accumulation via suppressing mTORC1 signaling pathway in hepatocytes

We next sought to delineate the mechanism underlying BMP4-inhibited hepatic steatosis. Using the PI3K/mTOR inhibitor PF-04691502, we found both inhibitors effectively inhibited oleic acid-induced lipid accumulation in mouse primary hepatocytes (Figure 2C). BMP4 was shown to effectively inhibit the expression of mTOCR1 signaling members, such as *S6k, Deptor, Pras40, Rptor, mTor,* and *Srebf1* at 36h and/or 72h after Ad-B4 infection, while transiently up-regulating the expression of *Lipin1* at 36h after Ad-B4 infection (Figure 2D). Furthermore, through Western blotting analysis, we confirmed that BMP4 down-regulated the expression of DEPTOR, S6K, p-S6K and SREBF1, while up-regulating the expression of LIPIN1 at 72h (Figure 2E).

### Exogenous BMP4 suppresses hepatic triglyceride/lipid accumulation by up-regulating hepatic lipid turnover *in vivo*

To assess whether recombinant adenoviral vectors can mediate a sustained expression of transgenes via direct intrahepatic injection, we injected CsCl gradient-purified Ad-FLuc and Ad-GFP control viruses via transdermal intrahepatic injection, followed by Xenogen optical imaging after injection. We found the FLuc signals were readily detected at least 5 days after injection (Supplementary Figure 1C). To minimize potential toxicities, the high titered Ad-B4 and Ad-GFP used for intrahepatic injections were purified through CsCl gradient ultracentrifugation as described [23, 24], and Ad-B4-mediated expression of BMP4 was further confirmed by Western blotting (Supplementary Figure 1B a and b).

**Figure 3:**
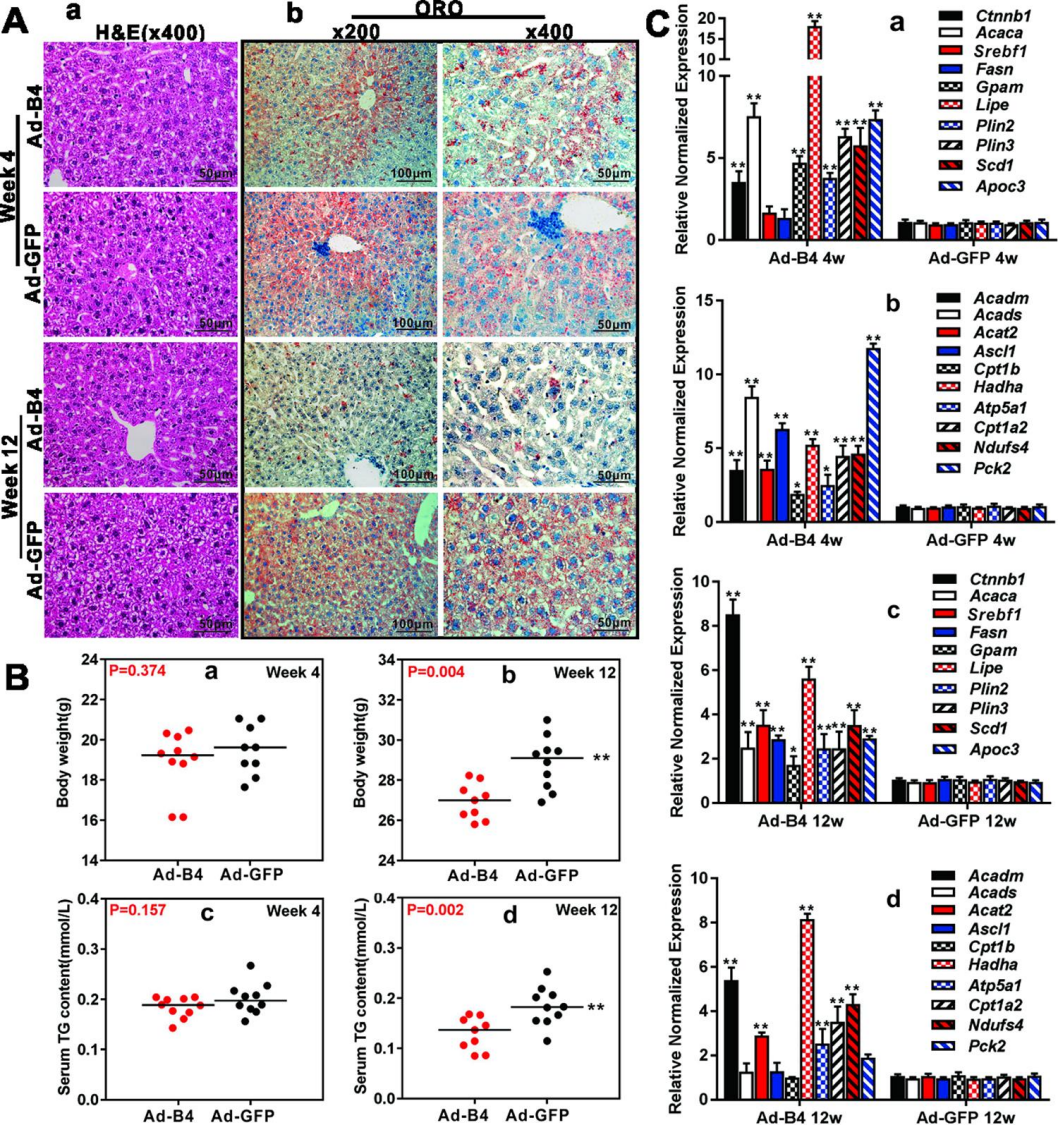
**Exogenous BMP4 decreases the body weights, inhibits serum and hepatic triglyceride accumulation *in vivo*.** (**A**) Ad-B4 or Ad-GFP was intrahepatically injected into 4-week old mice. The mice were sacrificed at weeks 4 and 12, and the retrieved liver tissue was subjected to H & E staining *(a)* and ORO staining *(b)*. (**B**) The mouse body weights at weeks 4 and 12 *(a and b)*, and serum total triglyceride (TG) *(c and d)* at weeks 4 and 12 were measured respectively. (**C**) Total RNA was isolated from the liver tissue of the mice injected with Ad-B4 or Ad-GFP at weeks 4 and 12 respectively, and TqPCR analysis was carried out to detect the expression of triglyceride synthesis and storage related genes *(a and c)* and triglyceride breakdown related genes *(b and d).* All samples were normalized with *Gapdh*. Relative expression was calculated by dividing the relative expression values (i.e., gene/*Gapdh*) in “**” p < 0.01, “*” p < 0.05, Ad-B4 group vs. Ad-GFP group. Each assay condition was done in triplicate, and representative images are shown or indicated by arrows.

We next tested the effect of exogenous BMP4 on hepatic lipid metabolism *in vivo*. Direct intrahepatic injections of Ad-B4 or Ad-GFP did not cause any apparent histologic changes in mouse liver after 4 weeks, while hepatocytes became swelling and some hepatic steatosis-like changes were observed in the Ad-GFP injection group, but not in the Ad-B4 injection group at 12 weeks, as assessed by H & E staining (Figure 3A, a). Furthermore, ORO staining revealed that Ad-B4 injection significantly inhibited hepatic lipid accumulation at both weeks 4 and 12(Figure 3A, b). While no significant changes in the body weights and serum TG concentrations were observed at week 4(Figure 3B, a and b), Ad-B4 injections led to the decreases in body weights and serum TG concentrations at week 12 (Figure 3B, c and d). Quantitative analysis using TqPCR further showed that exogenous expression of BMP4 significantly up-regulated the expression of lipid synthesis and storage genes (Figure 3C, a and c) and lipid breakdown genes (Figure 3C, b and d) at both weeks 4 and 12, suggesting BMP4 may promote the turnover rate of hepatic lipids *in vivo*.

### Exogenous BMP4 blocks hepatic triglyceride/lipid accumulation and attenuates the development and progression of NAFLD *in vivo*

**Figure 4:**
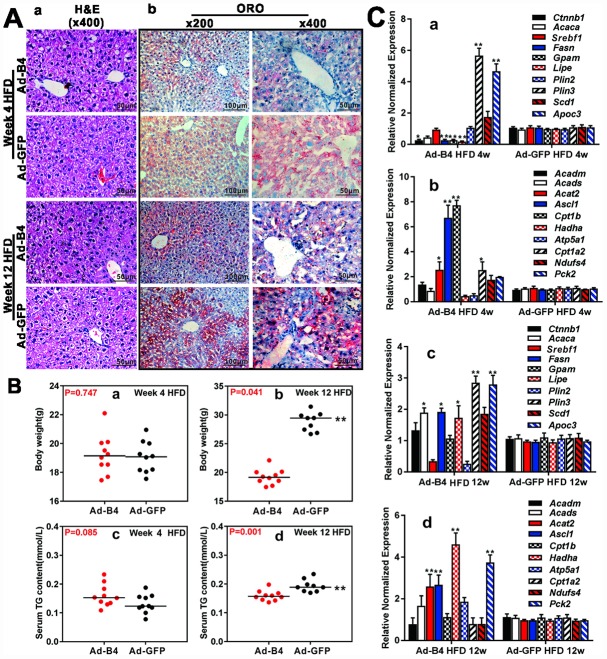
**Exogenous BMP4 alleviates lipid accumulation and inhibits the development and progression of a mouse model of NAFLD.** Ad-B4 or Ad-GFP were intrahepatically injected into the mice treated with HFD, and the mice were sacrificed at weeks 4 and 12 for following analyses. (**A**) The retrieved liver tissue was subjected to H & E staining *(a)* and ORO staining *(b)*. (**B**) The body weights at weeks 4 and 12 *(a and b)*, and serum total triglyceride (TG) at weeks 4 and 12 *(c and d)* were measured respectively. (**C**) Total RNA was isolated from the retrieved liver tissue of the HFD mice injected with Ad-B4 or Ad-GFP at weeks 4 and 12 respectively, and subjected to TqPCR analysis of the expression of triglyceride synthesis and storage related genes *(a and c)* and triglyceride breakdown related genes *(b and d).* All samples were normalized with *Gapdh*. Relative expression was calculated by dividing the relative expression values (i.e., gene/*Gapdh*) in “**” p < 0.01, “*” p < 0.05, Ad-B4 group vs. Ad-GFP group. Each assay condition was done in triplicate, and representative images are shown.

We further tested whether BMP4 could influence the disease process of NAFLD. When intrahepatic injections of Ad-B4 or Ad-GFP were initiated concurrently with the commencement of HFD-induced NAFLD in mice, hepatic steatosis-like changes were observed in the Ad-GFP injection group at 12-week time point, based on H & E staining while we did not observed any apparent histologic changes of the liver in both Ad-B4 and Ad-GFP groups at 4-week time point (Figure 4A, a). ORO staining revealed that Ad-B4 injection significantly suppressed lipid accumulation in the liver, compared with that of the Ad-GFP injection group, at both 4-week and 12-week time points (Figure 4A, b). Body weight measurements indicated that Ad-B4 injection significantly decreased the body weights at week 12 (Figure 4B, a and b), and there was a detectable decrease in serum TG levels in the Ad-B4 injection group at week 12 as well (Figure 4B, c and d). Furthermore, TqPCR analysis revealed that BMP4 injections effectively affected the expression of lipid synthesis and storage genes (Figure 4C, a and c) and lipid breakdown genes (Figure 4C, b and d) at both weeks 4 and 12, suggesting that exogenous BMP4 may significantly impact the dysregulated lipid metabolism during the development of NAFLD.

### Exogenous BMP4 inhibits hepatic steatosis and the development/progression of NAFLD by suppressing the mTORC1 signaling pathway *in vivo*

**Figure 5:**
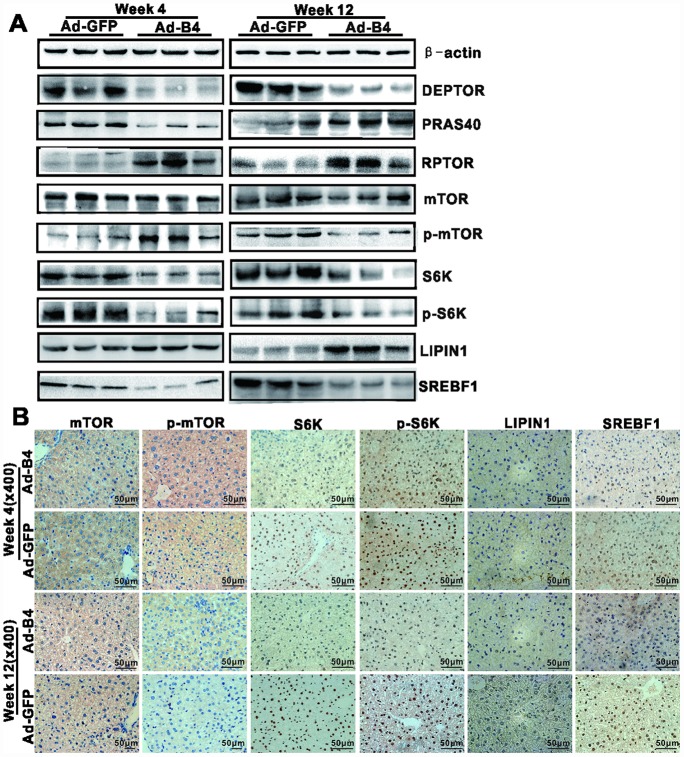
**BMP4 down-regulates the mTORC1 signaling pathway in mouse liver.** The liver samples prepared in Figure 3 were used for the following assays. (**A**) Total cell lysate was prepared from the retrieved liver samples and subjected to Western blotting analysis of the expression of the members of mTORC1 signaling pathway. (**B**) The retrieved liver samples were paraffin-embedded, sectioned and subjected to IHC staining to detect the expression of the members of mTORC1 signaling pathway and lipid metabolism. Each assay condition was done in triplicate, and representative images are shown.

We explored the underlying mechanism of BMP4 action and analyzed the expression of mTOR signaling components in the liver samples isolated from the normal mice injected with Ad-B4 or Ad-GFP. Using Western blotting analysis, we found that intrahepatic injection of Ad-BMP4 decreased the expression of DEPTOR, PRAS40, S6K, p-S6K and SREBF1, and up-regulated the expression of RPTOR, p-mTOR, LIPIN1 at week 4 (Figure 5A). Similarly, BMP4 injections decreased the expression of DEPTOR, p-mTOR, S6K, p-S6K and SREBPF1 and up-regulated the expression of the PRAS40, RPTOR, LIPIN1 at week 12 (Figure 5A). Furthermore, immunohistochemical staining showed that S6K and p-S6K significantly were down-regulated while LIPIN1 was up-regulated with increased nucleus staining by BMP4 both at weeks 4 and 12 (Figure 5B). Control IgG of liver tissue was shown in Supplementary Figure 1D, b.

**Figure 6:**
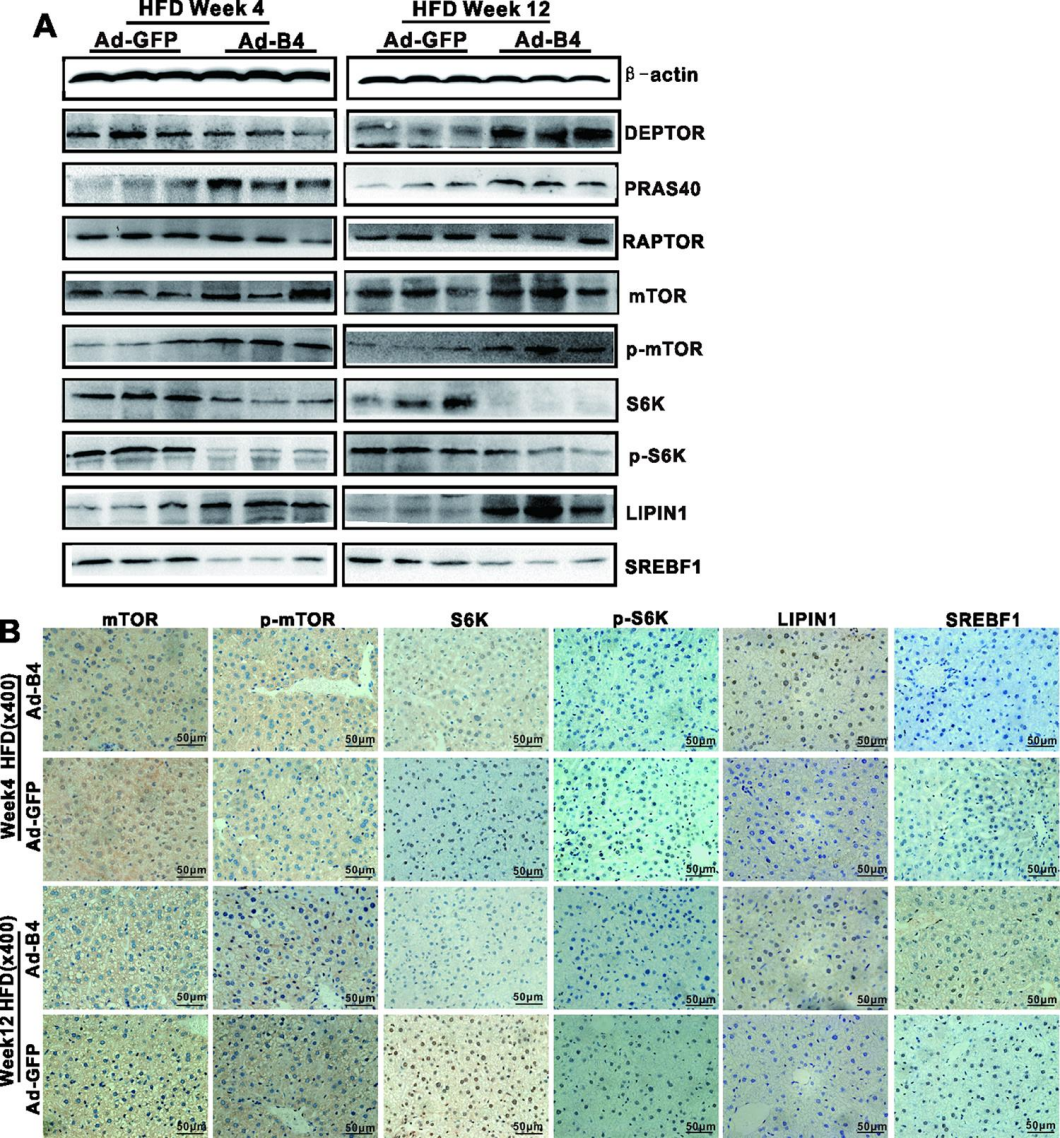
**Exogenous BMP4 attenuates the development and progression of NAFLD by suppressing the mTORC1 signaling pathway.** The liver samples prepared in Figure 4 were used for the following assays. (**A**) Total cell lysate prepared from the retrieved liver samples was subjected to Western blotting analysis to detect the expression of the members of mTORC1 signaling pathway and lipid metabolism. (**B**) The retrieved liver samples were paraffin-embedded, sectioned and subjected to IHC staining to detect the expression of the members of mTORC1 signaling pathway and lipid metabolism. Each assay condition was done in triplicate, and representative images are shown

Lastly, we examined the effect of exogenous BMP4 on mTOR signaling in NAFLD. Western blotting analysis revealed that Ad-B4 injections decreased the expression of S6K, p-S6K and SREBF1, while increased the expression of LIPIN1 both at weeks 4 and 12 (Figure 6A). Furthermore, immunohistochemical staining showed that Ad-B4 injections significantly down-regulated S6K, p-S6K and SREBF1 while increasing LIPIN1 expression in nucleus both at weeks 4 and 12 (Figure 6B). Control IgG of liver tissue was shown in Supplementary Figure 1D, c.

**Figure 7:**
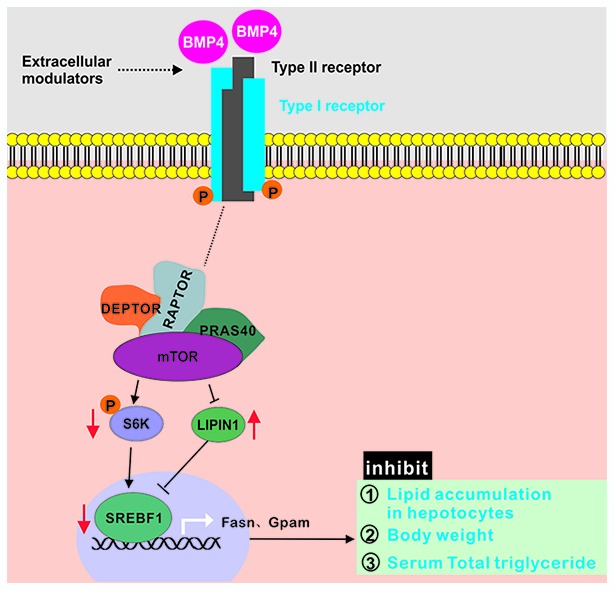
**BMP4 may suppress hepatic steatosis and alleviate the development and progression of NAFLD by inhibiting mTORC1 signaling.** BMP4 decrease the expression of S6K, p-S6K and SREBF1, while increase the expression of LIPIN1.

Collectively, these results strongly suggest that exogenous BMP4 may suppress hepatic steatosis and alleviate the development and progression of NAFLD by inhibiting mTORC1 signaling (Figure 7).

## DISCUSSION

In this study, we investigated the direct effect of BMPs on hepatic metabolism and their potential links with NAFLD. We found that BMP4 expression was elevated during the development of mouse model of NAFLD, whereas exogenous BMP4 was shown to inhibit hepatic triglyceride/lipid accumulation by promoting lipid turnover and by suppressing the mTORC1 signaling pathway. These results also suggest that the elevated BMP4 expression in NAFLD may be caused by a feedback inhibition mechanism.

BMPs, mostly BMP2, BMP4 and BMP7, have been shown to play important roles in regulating adipogenesis, thermogenesis, and energy metabolism in adipocyte progenitor cells and adipose tissue, and/or at whole animal levels [14–20]. An early study revealed that BMP2 induced a phenotypic change of C3H10T1/2 stem cells from the parental fibroblast to adipocytes and osteoblasts [25], while TGF-β and activin A were shown to inhibit adipocyte development [26]. BMP4 was shown to drive pluripotent C3H10T1/2 stem cells to adipocyte lineage [27], which requires functional BMP/Smad signaling pathway [28].

Forced expression of BMP4 in white adipocytes reduced the mass of white adipocyte tissue (WAT) and the size of white adipocytes in mice, with an increased number of a white adipocyte cell types with brown adipocyte characteristics [16], which closely correlated with increased energy expenditure, improved insulin sensitivity, and protection against diet-induced obesity and diabetes [16]. Furthermore, silencing the BMP antagonist GREM1 and/or adding BMP4 during white adipogenic differentiation were shown to reactivate beige/brown markers [29]. It was shown that selective brown adipose tissue overexpression of *Bmp4* in mice induced a shift from a brown to a white-like adipocyte phenotype [17], suggesting that Bmp4 may be an important factor in the context of obesity and type 2 diabetes. Similarly, increased circulating BMP4 in mature mice prevented obesity and insulin resistance, and promoted subcutaneous WAT browning, leading to increased energy expenditure [19].

Nonetheless, it remains to be fully determined whether BMP-regulated lipid metabolism affects the development and/or progression of obesity, metabolic syndrome and NAFLD. A small cohort study showed that serum BMP4 levels were significantly increased in individuals with obesity or metabolic syndrome [30]. Several BMPs and BMP receptors were implicated in obesity-related traits in humans [26]. Genetic variants of BMP receptor 1A gene (BMPR1A) were associated with human obesity [31]. As essential for BMP signaling BMP receptor 2 (BMPR2) was implicated in adipogenesis and pathophysiology of obesity [32]. Interestingly, intra-cerebroventricular administration of BMP7 was shown to ameliorate the HFD-associated metabolic complications, suggesting that BMP7 may be explored as an attractive obesity therapeutic for diet-induced obesity and leptin-resistant conditions [14].. Rapamycin (mTOR), a kinase that is activated by anabolic signals, plays fundamental roles in regulating lipid biosynthesis and metabolism. The mTOR kinase nucleates two large protein complexes named mTOR complex 1 (mTORC1) and mTOR complex 2 (mTORC2) [35]. Both mTORC1 and mTORC2 share four protein components, including the TOR kinase, DEP domain-containing mTOR-interacting protein (DEPTOR) and mammalian lethal with Sec13 protein 8 (mLST8), while regulatory-associated protein of mTOR (RAPTOR) and proline-rich AKT substrate 40 kDa (PRAS40) are specific to mTORC1 [35, 36]. mTORC1 promotes protein synthesis and lipid synthesis, which rely on the phosphorylation of mTORC1 substrates, including ribosomal S6 kinase 1 (S6K1), eukaryotic translation initiation factor 4E (eIF4E)-binding proteins 1 and 2 (4E-BP1/2), UNC-5 like autophagy activating kinase (ULK1), and transcription factor EB (TFEB) [35, 37]. Hepatic Lipogenesis is catalyzed by the rate-limiting enzymes acetyl-CoA carboxylase (ACC) and fatty acid synthase (FAS), both of which are transcriptionally controlled by various transcriptional regulators in response to nutrients and hormones, including sterol response element-binding protein (SREBP) family members, carbohydrate-responsive element binding protein (ChREBP), and nuclear receptors (PPARγ, FXR, and LXR) [38, 39]. mTORC1 enhances lipogenesis via the positive regulation of SREBPs in an S6K1-dependent and S6K1-independent manner [40]. SREBPs belong to the family of basic helix-loop-helix-leucine zipper (bHLH-Zip) transcription factors, among which SREBP1c and SREBP2 are the major isoforms expressed in the liver [41]. The mechanism of S6K1-dependent activation of SREBP is unclear, while the S6K1-independent activation of SREBP involves inhibition and phosphorylation of CRTC2 (CREB regulated transcription coactivator 2) and Lipin-1(a phosphatidic acid phosphatase required for glycerolipid biosynthesis) [41–43]. Our studies may provide a possible mechanistic link between BMP signaling and mTOR pathway in lipid metabolism. In summary, we demonstrated that exogenous BMP4 inhibited the hepatic steatosis and alleviated the development and progression of NAFLD in a mouse model. Mechanistically, BMP4 exerted these effects by promoting lipid turnover through up-regulating the genes involved in lipid metabolism, while suppressing mTORC1 signaling pathway. Thus, our findings strongly suggest that BMP4 may play an essential role in regulating hepatic lipid metabolism and the molecular pathogenesis of NAFLD. It is conceivable that manipulating BMP4 and/or mTORC1 signaling networks would lead to the development of novel therapeutics for obesity, metabolic syndrome, and NAFLD.

## MATERIALS AND METHODS

### Cell culture and chemicals

293pTP and RAPA cells were derived from HEK-293 cells as previously described [44, 45]. Primary hepatocyte were prepared from 4-week-old C57BL/6J mice (both genders) using the type I collagenase/liver perfusion protocol as described [46]. All above cells were maintained in complete DMEM supplemented with 10% fetal bovine serum (Lonsa Science SRL), 100 units of penicillin and 100 mg of streptomycin at 37°C in 5%CO_2_. Unless indicated otherwise, all chemicals were purchased from Sigma-Aldrich (St Louis, MO, USA), Thermo Fisher Scientific (Waltham, MA, USA), and/or Solarbio (Beijing, China).

### Construction, amplification and purification of recombinant adenoviruses

Recombinant adenoviruses Ad-B4 and Ad-FLuc were constructed by using the AdEasy technology [23, 47]. Briefly, the full-length coding regions of human BMP4 and firefly luciferase were PCR amplified and subcloned into an adenoviral shuttle vector, pAdTrack-CMV. The resultant vectors were used to generate recombinant adenoviral vectors, pAd-B4 and pAd-FLuc, through homologous recombination with an adenoviral backbone vector in bacterial BJ5183 cells, which were subsequently used to generate recombinant adenoviruses Ad-B4 and Ad-Fluc in 293pTP or RAPA cells. The resulting adenoviruses Ad-B4 and Ad-FLuc also co-express GFP as a marker for tracking infection efficiency as described [11, 48]. An analogous adenovirus expressing GFP only, Ad-GFP, was used as a mock control [49–51]. Polybrene (final concentration at 8μg/ml) was added to enhance adenoviral infection efficiency [52]. For the direct *in vivo* injection studies, the adenoviruses (Ad-B4, Ad-FLuc and Ad-GFP) were purified through CsCl gradient ultracentrifugation, followed by desalting dialysis immediately prior to use, as described [23, 53].

### Oil Red O (ORO) staining for triglyceride/lipid accumulation

ORO staining was carried out as described [11, 54]. Briefly, primary mouse hepatocyte was seeded in 24-well culture plates and treated with different conditions for 10 days. Alternatively, frozen sections from freshly prepared liver tissue samples were washed with PBS twice to remove embedding agents at room temperature (RT). Both cells and frozen sections were then fixed with 4% paraformaldehyde for 10min at RT, briefly incubated in 60% isopropanol, and then stained with freshly-prepared ORO solution for 15 min at RT, followed by multiple washes with PBS as described [11, 54]. The staining results were recorded under a bright field microscope (magnification, x200 for cells and x400 for tissue). Each assay condition was done in triplicate.

### Total RNA isolation of tissues/cells and touchdown-quantitative real-time PCR (TqPCR) analysis

Total RNA was isolated from both cells and freshly-prepared liver tissues by using the TRIZOL Reagent (Invitrogen, China) as described [55, 56]. Briefly, fresh mouse liver samples at different development stages (n=5, CD1 male, each time point) or from the NAFLD model were dissected out, minced, and ground in TRIzol Reagent. Similarly, primary mouse hepatocytes were treated with different conditions and lysed in TRIZOL Reagent for total RNA isolation. Total RNA was subjected to reverse transcription with hexamer and M-MuLV reverse transcriptase (New England Biolabs, Ipswich, MA). The cDNA products were used as qPCR templates. The gene-specific PCR primers were designed by using Primer3 Plus (Supplementary Table 1). TqPCR was carried out by using 2x SYBR Green qPCR Master Mix (Bimake, Shanghai, China) on a CFX-Connect unit (Bio-Rad Laboratories, Hercules, CA) as described [57, 58]. All TqPCR reactions were done in triplicate. *Gapdh* was used as the reference gene. Quantification of gene expression was carried out by using the 2-ΔΔCq method as described [59].

### Western blotting analysis

Western blotting assay was carried out as described [60, 61]. Briefly, subconfluent primary hepatocytes were infected with Ad-B4 and Ad-GFP for 72h. Cell lysates were prepared and subjected to SDS-PAGE, followed by electro-transferring to PVDF membranes, which were blocked and incubated overnight with the primary antibodies against β-ACTIN (1:5000-1:20000 dilution; Proteintech; Cat#60008-1-Ig), BMP4 (1:1000-1:3000 dilution; GeneTex; Cat#GTX100874), DEPTOR(1:100 dilution; Santa Cruz Biotechnology; Cat#sc-398169), PRAS40 (1:100 dilution; Santa Cruz Biotechnology; Cat#sc-517355), RPTOR (1:100 dilution; Santa Cruz Biotechnology; Cat#sc-81537), MTOR (1:1000-1:3000 dilution; GeneTex; Cat#GTX101557), p-MTOR (phospho Ser2448 1:1000-1:3000 dilution; GeneTex; Cat#GTX132803), S6K (1:1000-1:3000 dilution; GeneTex; Cat#GTX107562), p-S6K(phosphor S424 1:1000-1:3000 dilution; Abcam; Cat#ab131436), LIPIN1 (1:1000-1:3000 dilution; Abcam; Cat#ab181389) and SREBF1 (1:1000-1:3000 dilution; Proteintech; Cat#14099-1-AP). After being washed, the membranes were incubated with respective secondary antibodies (1:5000 dilution; ZSGB-BIG; Peroxidase-Conjugated Rabbit anti-Goat IgG or Peroxidase-Conjugated Goat anti-Mouse IgG; Cat#ZB-2306 or 2305). Immune-reactive signals were visualized by using the Enhanced Chemiluminescence (ECL) kit (Millipore, USA) on the Bio-Rad ChemiDoc Imager (Hercules, CA).

### Measurement of serum triglyceride (TG) in mice

At each endpoint, the animals were anesthetized and subjected to cardiac puncture to collect whole blood as described [62], followed by centrifugation at 800 x g, 10min, RT. The collected sera were used to determine triglyceride (TG) concentrations by using the Triglyceride Assay kit in microplate reader format (Single reagent GPO-PAP; Nanjing Jiancheng Bioengineering Institute, China).

### Establishment of the mouse model of non-alcoholic fatty liver disease (NAFLD)

The use and care of animals in the present study was approved by the Ethics Committee for Research and Experimental Animal Use of Chongqing Medical University (Chongqing, China; Certificate No: SCXK (YU)20070001). All animal experiments were performed in accordance with US National Institutes of Health Guide for the Care and Use of Laboratory animals [63]. The NAFLD model was established as described [64]. Briefly, 60 C57BL/6 (male, 4-week-old) were obtained from and housed at the Chongqing Medical University Experimental Animal Research Center. The mice were randomly divided into two groups (n=30 each): a high-fat diet (HFD) group fed with 45% fat diet (Medicience, Yangzhou, China) and a control group fed with10% fat diet (Supplementary Table 2). Ten mice from each group were sacrificed at weeks 10, 16 and 24, respectively. The retrieved liver tissue samples were either fixed with 4% paraformaldehyde for histologic and immunohistochemical analyses, or snap-frozen in liquid nitrogen for total RNA/protein isolation.

### H & E staining and immunohistochemical (IHC) staining

The retrieved mouse liver samples were fixed in 4% paraformaldehyde overnight, paraffin embedded, and sectioned. The sections were deparaffinized, rehydrated and subjected to H & E staining and IHC staining as described [50, 58, 65, 66]. Briefly, the sections were subjected to deparaffnization, followed by H & E. For IHC staining, the sections were subjected to deparaffnization, followed by antigen retrieval and immunostaining with antibodies against BMP4 (1:100-1:200 dilution; GeneTex; Cat#GTX100874), mTOR (1:100-1:200 dilution; GeneTex; Cat#GTX101557), p-mTOR (phospho Ser2448 1:100-1:200 dilution; GeneTex; Cat#GTX132803), S6K (1:100-1:200 dilution; GeneTex; Cat#GTX107562), p-S6K (phosphor S424 1:100-1:200 dilution; Abcam; Cat#ab131436), LIPIN1 (1:100-1:200 dilution; Abcam; Cat#ab181389), and SREBP1 (1:100-1:200 dilution; Proteintech; Cat#14099-1-AP). Control rabbit IgG was used as a negative control (1:200 dilution; Abcam; Cat#ab97051).

### Optical bioluminescence imaging of the mice with intrahepatic injection of Ad-FLuc

The *in vivo* use of recombinant adenoviruses was approved by the Ethics Committee for Research and Experimental Animal Use of Chongqing Medical University. To assess the duration of adenovirus-mediated gene expression in liver, we injected the CsCl gradient purified Ad-FLuc into mouse liver (4-week-old male, 10^10^ pfu/injection/mouse). Whole body optical imaging was performed at day 5 after injection by using D-Luciferin Potassium (Gold Biotechnology Inc.) as luciferase substrate and assessed with the Xenogen IVIS 200 Imaging System as described [67–70].

### Intrahepatic delivery of Ad-B4/Ad-GFP into mice

The *in vivo* use of recombinant adenoviruses was approved by the Ethics Committee for Research and Experimental Animal Use of Chongqing Medical University. Animals were obtained from and housed in the Chongqing Medical University Experimental Animal Research Center, and the experimental procedure was carried out as previously described [71]. Briefly, 40 male 4-week-old C57BL/6 mice were randomly divided into two groups (n=20 each) with normal diet fed: an Ad-B4 group (i.e., Ad-B4), and an Ad-GFP group (i.e., Ad-GFP). 40 male interpretation period C57BL/6 mice were randomly divided into two groups (n=20 each) with high fat diet (HFD) fed: a HFD treated with Ad-BMP4 group (i.e., Ad-B4 HFD), and a HFD fed with Ad-GFP group (i.e., Ad-GFP HFD). Intrahepatic injections of Ad-B4 or Ad-GFP (10^10^ pfu in 30μl PBS/injection/animal) were initiated immediately after the mice were fed with normal diet or HFD, and were re-dosed every 5 days, based on optical imaging of the Ad-FLuc intrahepatic injection in C57BL/6 mice. Ten mice from each group were sacrificed at weeks 4 and 12, respectively. The retrieved liver tissue was either fixed with 4% paraformaldehyde or snap-frozen in liquid nitrogen for total RNA/protein isolation.

### Statistical analysis

All quantitative experiments were performed in triplicate and/or repeated three times. Data were expressed as mean ± standard deviation (SD). The Least Significant Difference (LSD) was performed following the one-way analysis of variance (ANOVA) analysis to determine significant differences between groups. A value of p < 0.05 was considered statistically significant.

## Supplementary Material

Supplementary Figure 1

Supplementary Tables
