# Correction for: Bone morphogenetic protein 4 (BMP4) alleviates hepatic steatosis by increasing hepatic lipid turnover and inhibiting the mTORC1 signaling axis in hepatocytes

**DOI:** 10.18632/aging.102759

**Published:** 2020-01-13

**Authors:** Qi Peng, Bin Chen, Hao Wang, Ying Zhu, Jinghong Wu, Yetao Luo, Guowei Zu, Jinyong Luo, Lan Zhou, Qiong Shi, Yaguang Weng, Ailong Huang, Tong-Chuan He, Jiaming Fan

**Affiliations:** 1 Ministry of Education Key Laboratory of Diagnostic Medicine, and School of Laboratory Medicine, Chongqing Medical University, Chongqing 400016, China; 2 Molecular Oncology Laboratory, Department of Orthopaedic Surgery and Rehabilitation Medicine, The University of Chicago Medical Center, Chicago IL 60637, USA; 3 Clinical Epidemiology and Biostatistics Department, Department of Pediatric Research Institute, Children’s Hospital of Chongqing Medical University, Chongqing 400014, China; 4 Key Laboratory of Molecular Biology for Infectious Diseases of The Ministry of Education of China, Institute for Viral Hepatitis, Department of Infectious Diseases, The Second Affiliated Hospital of Chongqing Medical University, Chongqing, China

**Keywords:** correction

**This article has been corrected: **The authors requested to replace Figures 1, 2 and 5. The authors uploaded the wrong images by mistake during the preparation of the article. These corrections do not change any of the conclusions of the publication in any means. The corrected Figures 1, 2 and 5 are provided below.

**Figure 1:**
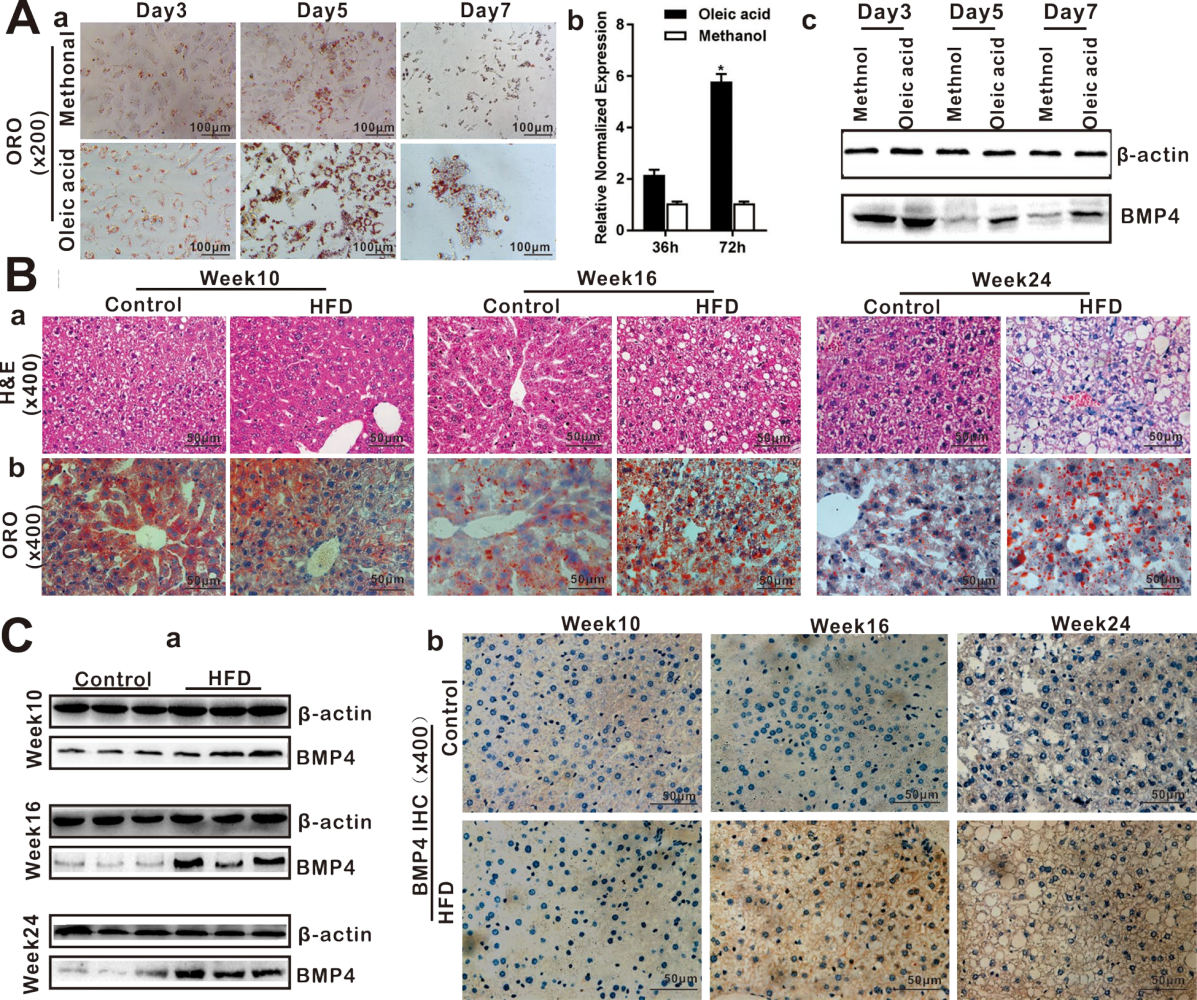
**BMP4 expression is elevated during Oleic acid-induced triglyceride/lipid accumulation in hepatocytes and in a mouse model of NAFLD. **(**A**)****BMP4 expression in Oleic acid-induced lipid accumulation. Primary mouse hepatocytes were stimulated with 0.05mM Oleic acid (methanol as a vehicle control). ORO staining was done at days 3, 5 and 7 respectively. Representative images are shown (*a*). Alternatively, total RNA was isolated at 36h and 72h post Oleic acid treatment and subjected to TqPCR analysis of Bmp4 expression. Relative expression was calculated by dividing the relative expression values (i.e., *Bmp4*/*Gapdh*) in “*” p < 0.05, Oleic acid group vs. methanol group (*b*). Total protein was isolated and subjected to Western blotting analysis of BMP4 expression at days 3, 5 and 7 post Oleic acid treatment (*c*). (**B**) Establishment of the mouse model of NAFLD. C57/B6 mice (4-week-old male, n=10 /time point/group) were fed with 45% high fat diet (HFD) or normal diet (Control), and sacrificed at weeks 10, 16 and 24, respectively. The retrieved liver tissue was subjected to H & E staining (*a*) and ORO staining (*b*). (**C**) BMP4 expression in mouse liver tissue of NAFLD. Total protein was isolated from the mouse liver tissue of the HFD and Control groups at weeks 10, 16 and 24 respectively, and subjected to Western blotting analysis of BMP4 expression. (*a*). IHC (immunohistochemical) staining of BMP4 expression was detected in the liver from the HFD and Control groups respectively (*b*). Each assay condition was done in triplicate, and representative images are shown or indicated by arrows.

**Figure 2:**
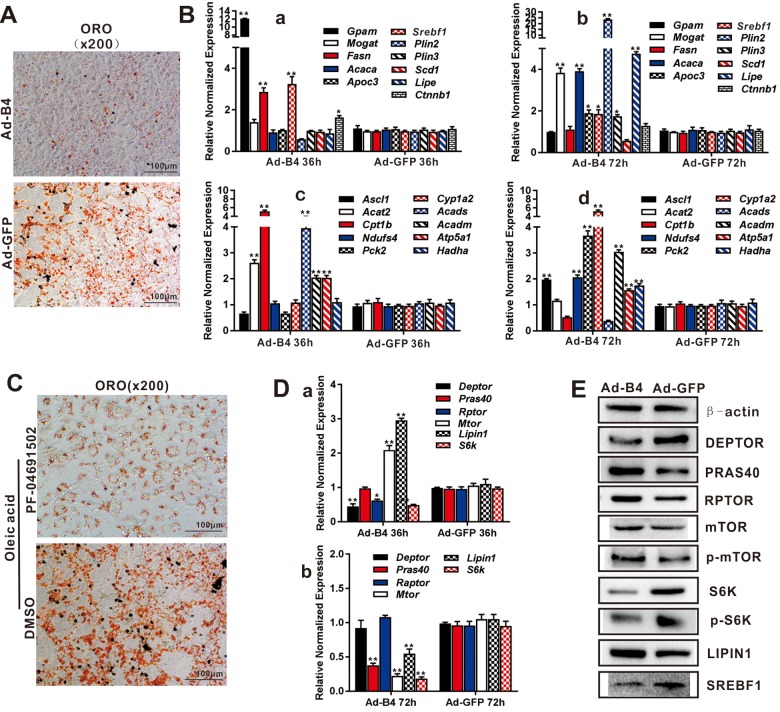
**BMP4 inhibits triglyceride accumulation through regulating the genes involved in lipid metabolism and members of mTORC1 signaling pathway in hepatocytes.** (**A**) Primary mouse hepatocytes were infected with Ad-B4 or Ad-GFP for 7 days, and subjected to ORO staining. (**B**) Primary mouse hepatocytes were infected with Ad-B4 or Ad-GFP for 36h and 72h. Total RNA was isolated and subjected to TqPCR analysis of the expression of the genes involved in triglyceride synthesis and storage (*a *and* b)* and triglyceride breakdown *(c *and* d)*. Relative expression was calculated by dividing the relative expression values (i.e., gene/*Gapdh*) in “**” p < 0.001, “*” p < 0.05, Ad-B4 group vs. Ad-GFP group. (**C**) Oleic acid (0.05mM)-induced hepatocytes were treated with 1nM PF-04691502 or DMSO for 7 days, and subjected to ORO staining. (**D**) Primary mouse hepatocytes were infected with Ad-B4 or Ad-GFP for 36h and 72h. Total RNA was isolated and subjected to TqPCR analysis of the expression of the members of mTORC1 signaling pathway (*a* and* b)*. Relative expression was calculated by dividing the relative expression values (i.e., gene/*Gapdh*) in “**” p < 0.01, “*” p < 0.05, Ad-B4 group vs. Ad-GFP group. (**E**) Primary mouse hepatocytes were infected with Ad-B4 or Ad-GFP for 72h, and total cell lysate was subjected to Western blotting analysis of the expression of the members of mTORC1 signaling pathway*.* Each assay condition was done in triplicate, and representative images are shown or indicated by arrows.

**Figure 5:**
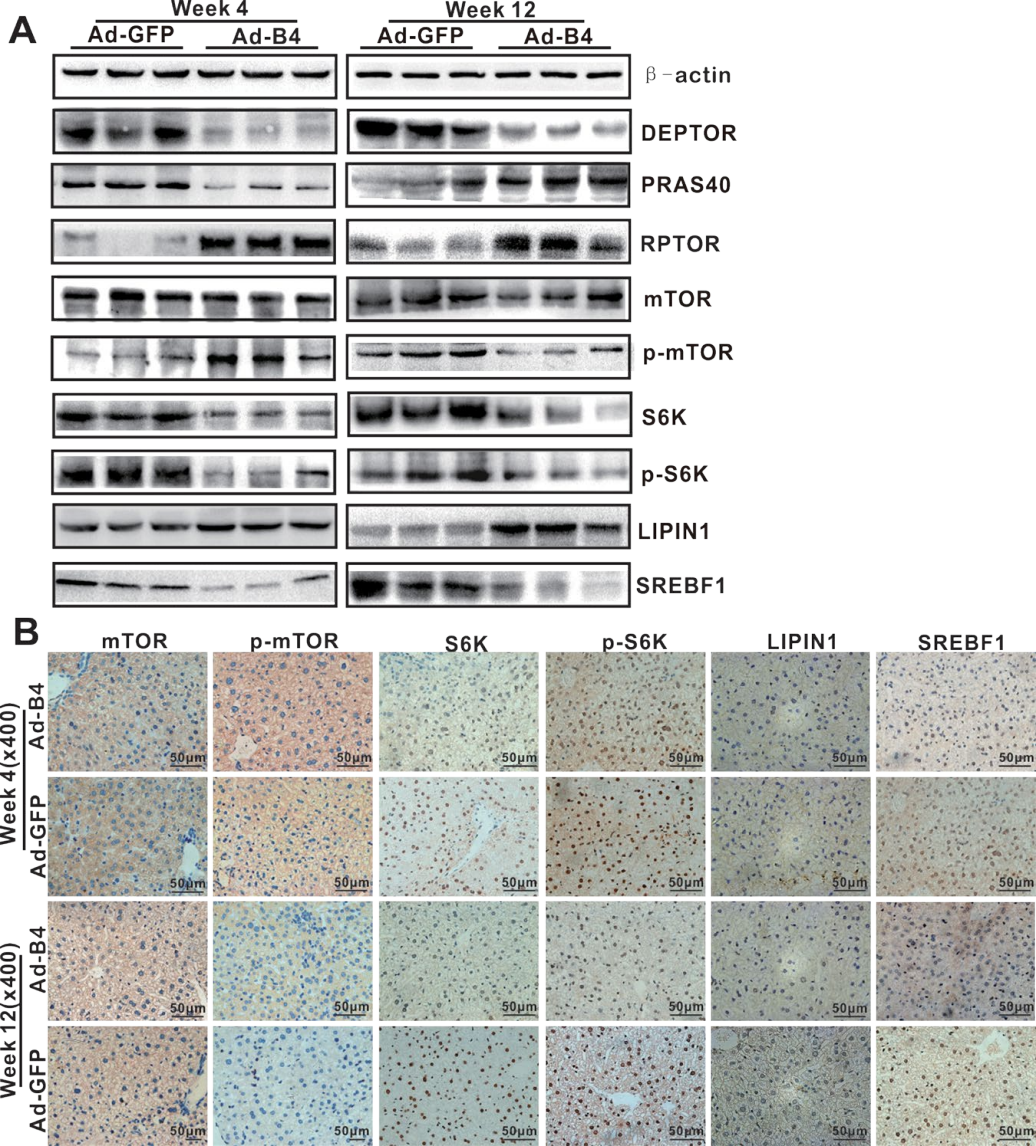
**BMP4 down-regulates the mTORC1 signaling pathway in mouse liver.** The liver samples prepared in Figure 3 were used for the following assays. (**A**) Total cell lysate was prepared from the retrieved liver samples and subjected to Western blotting analysis of the expression of the members of mTORC1 signaling pathway. (**B**) The retrieved liver samples were paraffin-embedded, sectioned and subjected to IHC staining to detect the expression of the members of mTORC1 signaling pathway and lipid metabolism. Each assay condition was done in triplicate, and representative images are shown.

Original article: Aging. 2019; 11:11520–11540.  . https://doi.org/10.18632/aging.102552

