# Supplementary material for: Bone morphogenetic protein 4 (BMP4) alleviates hepatic steatosis by increasing hepatic lipid turnover and inhibiting the mTORC1 signaling axis in hepatocytes
Source: Aging (Albany NY). 2019 Dec 12;11(23):11520–40. doi: 10.18632/aging.102552 (PMC6932923; doi:10.18632/aging.102552)
Supplement: Supplementary Figure 1 [file aging-11-102552-s002..pdf]

SUPPLEMENTARY FIGURE

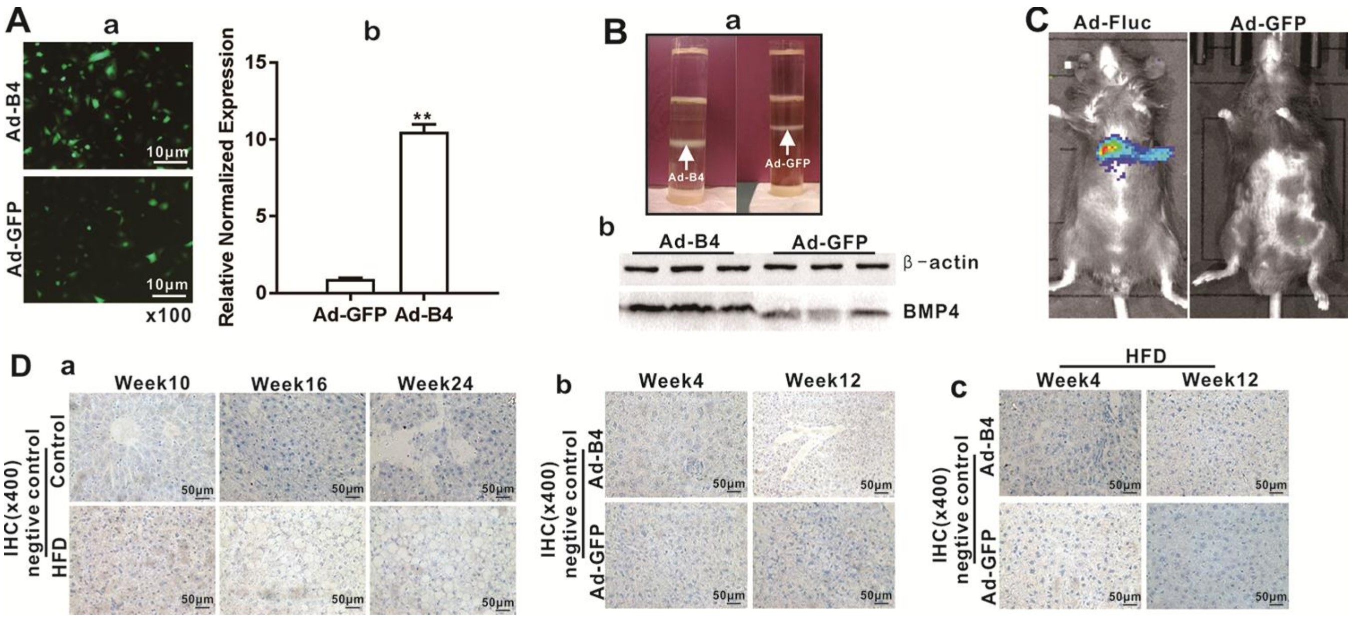

Supplementary Figure 1. (**A**) Ad-BMP4-mediated transgene expression in mouse hepatocytes. Ad-B4 or Ad-GFP were used to infect mouse primary hepatocytes isolated from 4-week-old mice for 48h. Total RNA was isolated for TqPCR analysis of the expression of human BMP4. Relative expression was calculated by dividing the relative expression values (i.e., gene/*Gapdh*) in "**" p < 0.01, Ad-B4 group vs. Ad-GFP group. (**B**) Ad-B4-mediated transgene expression post intrahepatic injections. High titer recombinant adenoviruses Ad-B4 and Ad-GFP were purified via CsCl gradient ultracentrifugation (***a***). The desired virus bands are indicated by arrows. The 4-week-old mice were subjected to the intrahepatic injection of Ad-B4 or Ad-GFP (10^{10} pfu in 30μl PBS/injection/animal, n=3 each virus) and sacrificed after 5 days. The retrieved liver samples were subjected to Western blotting to detect BMP4 expression*(**b**)*. (**C**) Adenovirus-mediated transgene expression lasts more than 5 days post intrahepatic injection. The CsCl gradient purified Ad-FLuc and Ad-GFP were intrahepatically injected into 4-week-old mice. The mice were subjected to optical bioluminescence imaging with a luciferin substrate at different time points after adenovirus administration. Representative imaging results at day 5 of intrahepatic injection are shown. (**D**) Paraffin sections of liver samples were subjected to IHC staining, stains without primary antibody were used as negative controls. The liver samples prepared in Figure 1 ***(a)***, The liver samples prepared in Figure 4 ***(b)***, The liver samples prepared in Figure 4 ***(c)***.
