# Supplementary material for: Bone morphogenetic protein 4 (BMP4) alleviates hepatic steatosis by increasing hepatic lipid turnover and inhibiting the mTORC1 signaling axis in hepatocytes
Source: Aging (Albany NY). 2019 Dec 12;11(23):11520–40. doi: 10.18632/aging.102552 (PMC6932923; doi:10.18632/aging.102552)
Supplement: Supplementary Tables [file aging-11-102552-s001..pdf]

SUPPLEMENTARY TABLES

Supplemental Table 1. List of TqPCR Primers.

Gene	Forward primer	Reverse primer	Accession No.
mouse Gapdh	ACCCAGAAGACTGTGGATGG	CACATTGGGGGTAGGAACAC	NM_008084
mouse Bmp4	GCGAGCCATGCTAGTTTGA	AAGTGTCGCCTCGAAGTCC	NM_001316360.1
human BMP4	GGGAAAGGGGCTTCCACC	TTTGGCTGCTTCTCCCGG	NM_001202.5
mouse Gpam	TTCCTGCACGCCACAGAG	TCTCGCCAGCCATCCTCT	NM_001356285.1
mouse Mogat1	CCTTCCTCCTGCTCGTGC	GCCAGCTTTGGACCCAGT	NM_026713.3
mouse Fasn	CTCGGCTCGATGGCTCAG	GGGATGTGCCACAGTGCT	NM_007988.3
mouse Acaca	GTGTTTGCTGGCCAGTGC	TGCCCGTGTGAAGCTCAG	NM_133360.2
mouse Apoc3	ACCCAGCCATCTAGCCCA	ACAGAGCCCAGCAGCAAG	NM_001289755.1
mouse Srebf1/Srebp	GACCCTACGAAGTGCACACA	GTGGCCTAGTCACAGGTTCC	NM_001313979.1
mouse Plin2	GAACCAGCCAACGTCCGA	TCCTTTCCACGCTGCCAG	NM_007408.3
mouse Plin3	GAGCATGTGTGTGGCCCT	CTCTGGCTATGCGCTCCC	NM_025836.3
mouse Scd1	TGCCGCGCATCTCTATGG	AGTTGTGGAAGCCCTCGC	NM_009127.4
mouse Lipe	CGTGCCAGCCACAACCTA	TGCAGGAAGTCAGCGGTG	NM_001039507.2
mouse Ctnnb1	TGCAGCTTCTGGGTTCCG	GCACAGATGGCAGGCTCA	NM_001165902.1
mouse Ascl1	GGAAGCAGGATGGCAGCA	GCCCCTGTAGGTTGGCTG	NM_008553.4
mouse Acat2	GCATGACAGCCACCACCT	GCTGTCCACCTGGGTTCC	NM_009338.3
mouse Cpt1b	GGAGGTGGCTTTGGTCCC	GGCGGATGTGGTTCCCAA	NM_009948.2
mouse Ndufs4	GGCGAAGGGCAATGGCTA	GCTGTGTGTCCCGAGTCT	NM_010887.2
mouse Pck2	GCTTACGTGGTGGCCAGT	CACGTGGCCAATCAGGGT	NM_028994.2
mouse Cyp1a2	CCAGTCAGCCAGGTGGTG	CTCTTGAGGGCCGGGTTG	NM_009993
mouse Acads(Bcl-1)	CACGCTGGGCAAGAAGGA	TGCCAATGCGACCCATGT	NM_007383.3
mouse Acadm	CGGAGGCAGCAGATCGAG	CTTGCGGGCAGTTGCTTG	NM_007382.5
mouse Atp5a1	GCACGGGCTGAGGAATGT	CCAACAGCTCCTCGCCAA	NM_007505.2
mouse Hadha	ACCACAGGCTTCGGCTTC	GCGACCCAAGAAGCCCTT	NM_130826.2
mouse Deptor	GTCAGCCCCAGCAAGGAG	TGGGGTTGCAGAGCACAG	NM_001037937.3
mouse Pras40/Akt1s1	GGCCAGGGAGGATGAGGA	TGTGCTCTCCGGGTCTGA	NM_001253920.1
mouse Rptor	ACGTGCGCATTGTCAGGA	CGGATCGAGCCATCACCC	NM_001306081.1
mouse Mtor	AAGGACCTCACGCAAGCC	ATTGGCTGGTTGGGGTCG	NM_020009.2
mouse Lipin1	AACGGAGCCGACACCTTG	GCTCCGTTGTCACTGGCT	NM_001130412.1
mouse Rps6kb1/S6k	CCTAGCGCCTGACTTCCG	CCCCCTCCTCCAGCTCAT	NM_001114334.2

Supplemental Table 2. Formula and fatty acid composition of control (10% fat) and high fat (45% fat) diets.

	MD10% Fat	MD45% Fat
**Energy Composition**	100	100
Protein	20	20
Carbohydrate	70	35
Fat	10	45
**Composition of fatty acid**	100	100
Saturated (%)	28.7	40.3
Monounsaturated (%)	32.7	40.4
Polyunsaturated (%)	38.6	19.3
**Type of fat (gm)**	45	202.5
Lard	20	177.5
Soybean Oil	25	25
**Fatty acid profile (gm)**	42.2	190.6
C2, Acetic	0	0
C4, Butyric	0	0
C6, Caproic	0	0
C8, Caprylic	0	0
C10,Capric	0.0	0.1
C12,Lauric	0.0	0.1
C14,Myristic	0.3	2.1
C15	0.0	0.2
C16,Palmitic	7.9	47.1
C16:1,Palmitoleic	0.4	3.4
C16:2	0	0
C16:3	0	0
C16:4	0	0
C17	0.1	0.6
C17:1	0	0
C18,Stearic	3.8	26.5
C18:1,Oleic	13.4	73.5
C18:2,Linoleic	14.6	32.4
C18:3,Linolenic	1.4	2.1
C18:4, Stearidonic	0	0
C20,Arachidic	0.0	0.2
C20:1	0.0	0.1
C20:2	0.1	0.4
C20:3	0.0	1.1
C20:4,Arachidonic	0.1	0.7
C20:5, Eicosapentaenoic	0	0
C21:5	0	0
C22, Behenic	0	0
C22:1, Erucic	0	0
C22:4, Clupanodonic	0	0
C22:5,Docosapentaenoic	0.0	0.2
C22:6, Docosahexaenoic	0	0
C24, Lignoceric	0	0
C24:1	0	0
